# Metastatic mucoepidermoid carcinoma to the pleura: a case report

**DOI:** 10.1186/s13256-022-03285-y

**Published:** 2022-02-16

**Authors:** Simran Mashiana, Ernesto Martinez Duarte

**Affiliations:** 1 Department of Pathology and Laboratory Medicine,, University of Nebraska Medical Center, Omaha, NE USA; 2 Department of Pathology, Immunology and Laboratory Medicine, University of Florida College of Medicine, 1600 SW Archer Road, Gainesville, FL 32610 USA

**Keywords:** Mucoepidermoid, Carcinoma, Metastatic, Pleura

## Abstract

**Background:**

Mucoepidermoid carcinoma is the most common malignant neoplasm arising from the salivary glands (Ali *et al.* in J Ayub Med Coll Abbottabad 20(2): 141-2, 2008, Xi *et al.* in World J Surg Oncol 10: 232, 2012). When arising from anatomic sites other than the salivary glands it can be a diagnostic challenge. Primary and metastatic mucoepidermoid carcinoma from and to the pleura are extremely rare entities that are frequently misdiagnosed as adenocarcinoma, adenosquamous carcinoma, or squamous cell carcinoma (Xi *et al.* in World J Surg Oncol 10: 232, 2012).

**Case presentation:**

We describe an unusual case of a 64-year-old Caucasian female patient with metastatic high-grade mucoepidermoid carcinoma to the pleura, morphologically resembling squamous cell carcinoma. Molecular studies of both the parotid gland and pleural tumors helped prove the metastatic nature of the pleural lesion.

**Conclusions:**

Metastatic mucoepidermoid carcinoma to the pleura is a rare entity, frequently misdiagnosed as squamous cell carcinoma. Differentiating between a lung primary and a metastatic disease has treatment implications and prognostic significance for the patient. When morphologic and immunophenotypic overlap exists, molecular testing can help distinguish mucoepidermoid carcinoma from other neoplasms.

## Introduction

Mucoepidermoid carcinoma (MEC) is the most common malignant neoplasm arising from the salivary glands [1, 2]. It frequently affects the parotid gland, followed by the submandibular, sublingual, and minor salivary glands [3]. It can also arise from the nasal cavity, bronchial wall, lungs, and pleura [3–6].

MECs are composed of mucinous, epidermoid, and intermediate cells in various degrees in an admixed solid and cystic architecture [6, 7]. High-grade mucoepidermoid carcinoma comprises solid sheets of epidermoid and clear cells with necrosis, nuclear pleomorphism, increased mitotic activity, and, rarely, focal keratinization [8, 9].

When arising in areas other than the salivary glands, it can be challenging and commonly misdiagnosed as adenocarcinoma, squamous cell carcinoma, or adenosquamous carcinoma [2].

## Case presentation

**Fig. 1:**
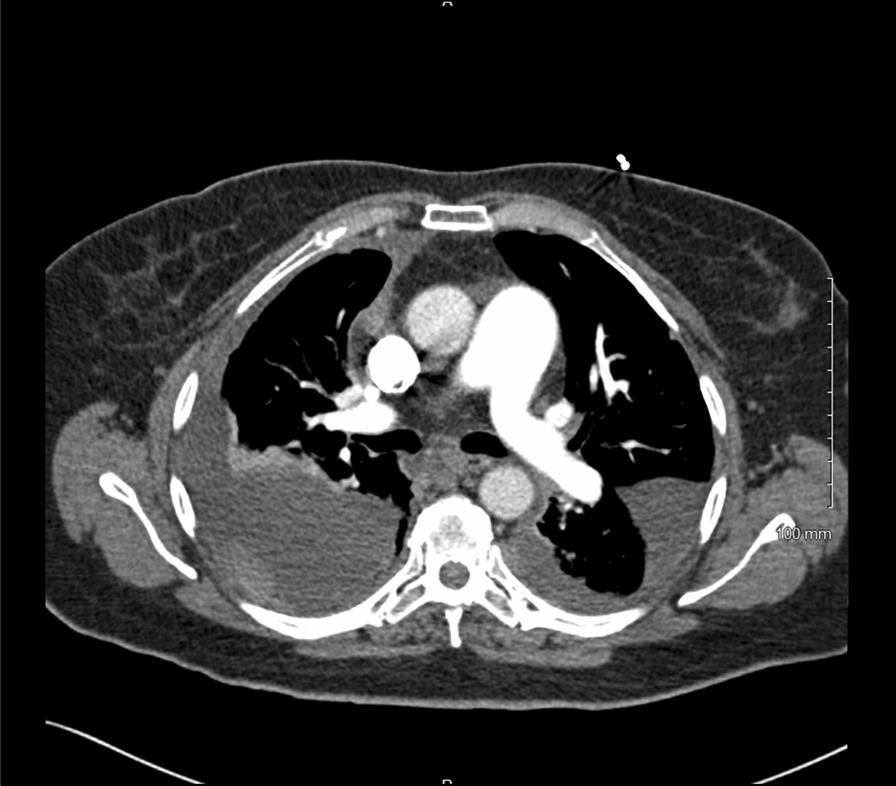
Computed tomography of the chest showing the large bilateral pleural based nodularities

A 64-year-old Caucasian female with a past medical history of asthma, congestive heart failure, hypertension, and diabetes mellitus presented to the emergency department with worsening dyspnea and cough for 3 weeks, which showed no improvement with antibiotics. Computed tomography (CT) of the chest revealed a right lung nodule measuring 1.2 cm in the largest dimension, bilateral pleural effusions, and multiple pleural-based nodules. The largest pleural nodule in the right chest measured 5.8 cm and in the left chest measured 4.8 cm (Fig. 1). The findings of multiple nodularities involving both lungs and pleura were suspicious for metastatic disease.

**Fig. 2:**
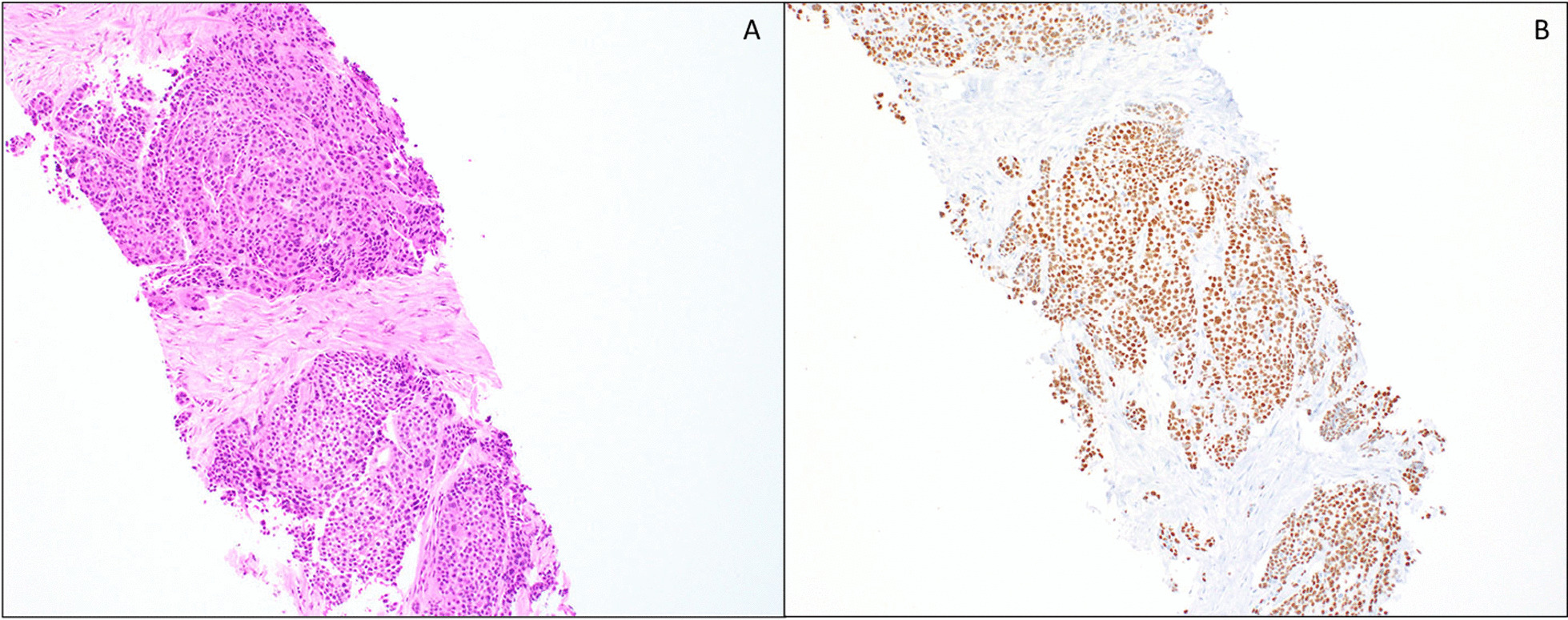
**A** Pleural nodule biopsy showing sheets of neoplastic cells with abundant eosinophilic cytoplasm (H&E 10×). **B** Pleural nodule biopsy p63 immunostain (10×)

Cytological examination of the pleural fluid showed reactive mesothelial cells and inflammatory cells with no evidence of malignancy. Subsequent biopsy of the right pleural nodule showed sheets of tumor cells with abundant eosinophilic cytoplasm and few scattered clear cells (Fig. 2A). Mucocytes were absent. The tumor cells were positive for p63 and negative for thyroid transcription factor 1 (TTF-1) by immunohistochemistry (Fig. 2B). A review of the patient’s past medical history was significant for a high-grade salivary gland neoplasm, which was resected and radiated 6 years prior at an outside facility. At this point, the differential diagnosis included squamous cell carcinoma of the lung with pleural involvement or metastasis from the previously resected salivary gland neoplasm.

**Fig. 3:**
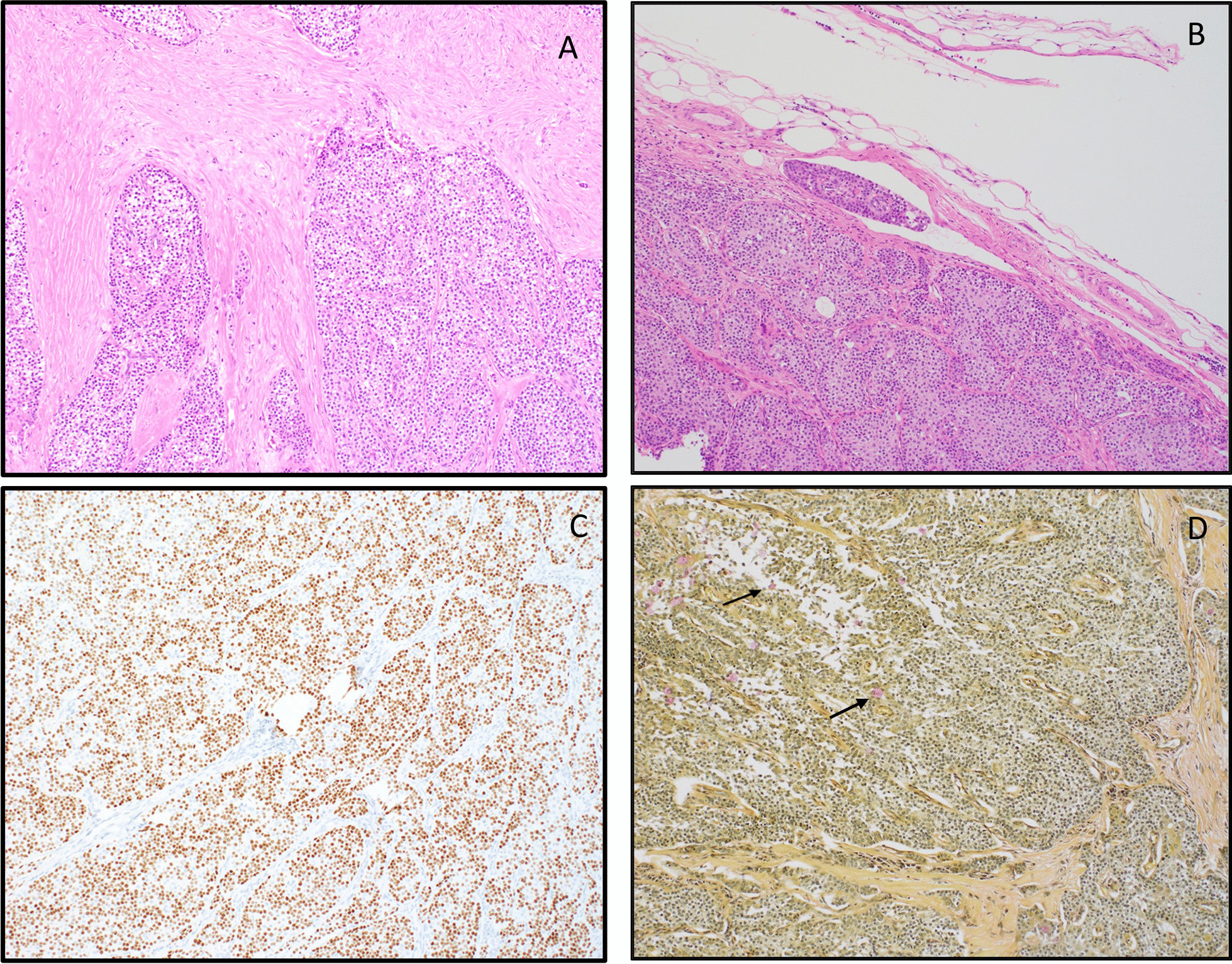
**A**, **B** Parotidectomy showing sheets of tumor cells with ample eosinophilic cytoplasm and lymphatic vessel invasion (H&E 10×). **C** Parotidectomy immunostain for p63 (10×). **D** Mucicarmine special stain (10×) highlights scattered mucocytes (arrows)

A review of the prior parotidectomy specimen and comparison of both lesions showed similar morphologic characteristics (Fig. 3A, B). The tumor cells from the previous resection were positive for cytokeratin 7(CK7), CAM5.2, p63, and CK5/6. Mucicarmine special stain highlighted a few mucocytes (Fig. 3C, D). The tumor cells were negative for vimentin, CDX2, estrogen receptor (ER), CK20, chromogranin, TTF-1, smooth muscle actin (SMA), synaptophysin, S100, and calponin. This tumor was diagnosed as high-grade salivary gland neoplasm at the referring institution without further subcategorization. Based on morphology and immunoprofile, the differential diagnosis included high-grade mucoepidermoid carcinoma and hyalinizing clear cell carcinoma.

Molecular studies performed on both specimens detected a CRTC1 (19p13) and MAML2 (11q21) fusion. The overall findings were thus consistent with metastatic mucoepidermoid carcinoma to the pleura.

After diagnosing metastatic MEC to the pleura, the patient became severely short of breath and developed bilateral pleural effusions that persisted despite evacuation. She developed respiratory distress, could not be weaned off the ventilator, and passed away 2 months after the diagnosis.

## Discussion

Primary salivary gland-like tumors of the lung and pleura are rare. They represent less than 1% of all lung and primary pleural neoplasms [5]. Primary mucoepidermoid carcinoma (MEC) tops the list, followed by adenoid cystic carcinoma [5]. MEC of the lung is typically centrally located, as they arise from mucous glands of the proximal tracheobronchial tree. The majority of these tumors are low grade and are more common in females than males [5, 10]. Primary pleural neoplasms are commonly mesotheliomas or adenocarcinomas, with salivary gland-like neoplasms being a rare occurrence. Moran and Suster theorize that since there are no salivary gland structures in the pleura, these neoplasms most likely arise from ectopic salivary gland tissue entrapped in the pleura [5]. Another important differential diagnosis to keep in mind when the tumor is pleural-based is solitary fibrous tumor. Outside these instances, any other primary disease of the pleura represents a rarity [5, 11]. As such, metastatic MEC is an infrequent occurrence, with, to our knowledge, only one other case described and presented at a meeting in 2012 by Jimenez and Singh [12].

Primary MECs of the lung and pleura are typically low-grade tumors, representing 0.1–0.2% of all primary pulmonary malignancies, affecting a younger patient population than its primary salivary gland counterpart [5, 11, 13].

In the salivary glands, they represent the most common malignancy, representing 10–25% of all malignant tumors in that region [8, 14–17]. They commonly arise from the parotid gland [18], followed by the submandibular gland and minor salivary glands (palate or buccal mucosa) [19–21].

Clinically, MEC can be asymptomatic or present as a solitary, painless, slow-growing mass. Less commonly, MEC presents with pain, facial numbness, paralysis, or respiratory symptoms such as dyspnea, cough, chest pain, and mucopurulent expectoration depending on location, size, extension, and compressive effects over underlying nerves. Pulmonary MEC usually present as endobronchial tumors with a polypoid growth, obstructing the bronchial lumen [3, 22–25].

MEC has a variable mucinous, epidermoid (squamoid), and intermediate cell component, forming variably cystic spaces [3, 7, 26]. The mucocytes can be arranged in nests or scattered as single cells within the tumor as intracytoplasmic mucin. Intermediate cells are large and polygonal, frequently with a clear cytoplasm found in nests and sheets, and it can be the predominant cell population, imparting a clear look under the microscope [3]. The cystic spaces are characteristic of low- to intermediate-grade neoplasms, often containing mucin with occasional papillary projections [3, 22]. Epidermoid cells form nests or can present as scattered polygonal cells. Mucoepidermoid carcinomas are associated with a dense peritumoral lymphoid infiltrate, known as tumor-associated lymphoid proliferation (TALP). TALP is typically present at advancing tumor edges, with the occasional germinal center formation, and can be confused with metastatic disease to lymph nodes. In this respect, CAM5.2 can help in cases where TALP and lymph node metastasis are not clear. CAM5.2 is positive in extrafollicular reticulum cells within lymph nodes and negative in TALP [27].

A set histologic criterion exists to grade MEC, based on the percentage of solid/cystic component, perineural invasion, and mitotic activity, subdividing this neoplasm into low-, intermediate-, and high-grade [28, 29]. Low-grade mucoepidermoid carcinomas rarely metastasize. Perineural invasion and positive surgical resection margins can hinder the excellent prognosis of low-grade MECs [18, 28, 30]. Tumor grading helps predict outcome and management, with high-grade MECs having a higher recurrence rate and metastasis [3, 26].

Typical immunoprofile of MEC shows strong nuclear reaction for p63 in the epidermoid and intermediate cells [22]. CK5/6 is also positive in epidermoid cells [3]. Ki67 shows higher expression in highly proliferative lesions, is indicative of a high-grade tumor, and its overexpression indicates poor prognosis [31, 32]. HER2 also tends to be strongly expressed in high-grade lesions, and this reactivity might guide future therapies with targeted anti-HER2 drugs [32]. P16 is positive in up to 60% of tumors, with a higher expression in the glandular component than in the squamoid component, and is not related to transcriptionally active human papillomavirus (HPV) [33, 34].

Up to 65% of MECs are reported to show t(11;19) translocation [15, 28, 35]. This translocation fuses CREB-regulated transcription coactivator 1 (CRTC1) (exon 1 of gene 19p13) with mastermind-like gene family (*MAML2*) (exons 2–5 of the gene at 11q21) [36, 37]. This translocation is present in low- to intermediate-grade tumors. Other genetic alterations have been found, such as t(11;15) (q21;q26) translocation resulting in CRTC3/MAML2 gene fusion (5% of tumors), usually seen in younger patients. Translocation (6;22) (p21;q12) with EWSR1-POU5F1 gene fusion, seen in high-grade tumors as well as CDKN2A deletions, seen in more aggressive MAML2 fusion-positive tumors [38]. Aneuploid tumors show a higher recurrence rate and cervical lymph node involvement, with decreased survival [36–39].

Treatment for pulmonary and pleural MEC is complete resection and either lobectomy, decortication, or simple resection of the pleura’s focal tumor area [2, 40]. Prognosis is dependent on tumor grade but is not very clear since so few cases have been reported [2, 12]. High-grade tumors may require medical treatment in addition to surgical resection [41].

Because these tumors are more common in the salivary glands or trachea, it is essential to obtain a careful clinical history of previous head and neck surgery or tumors. Histomorphology of primary and metastatic disease is often very similar. Metastatic tumors are common to the lung parenchyma, whereas primary tumors of the respiratory tract are primarily centrally located. Immunohistochemistry and molecular features are identical in this case; hence the history of mucoepidermoid carcinoma of the head and neck area is of utmost importance.

Pleura is an uncommon site for metastasis from primary salivary gland mucoepidermoid carcinoma but should be part of the differential diagnosis. An extensive review of the patient’s past medical history plays a crucial role in correctly classifying these lesions as primary or metastatic. Differential diagnoses should include primary squamous cell carcinoma, adenosquamous carcinoma, mesothelioma, and the pleura’s rare primary mucoepidermoid carcinoma.

Prognostically, grade is one of the most important factors, followed by the pathologic and clinical stages. Low-grade tumors rarely metastasize, with 95% disease-specific survival (DDS) of 5 years. High-grade tumors metastasize in 55–80% of cases, with 65% DSS of 5 years [42, 43]. Positive surgical resection margins are predictive of recurrence [34, 44]. Negative predictive factors include high-grade tumors (mitoses of more than 4/10 high-power field (HPF) and necrosis), nuclear pleomorphism, focal keratinization, desmoplasia, and lymph node metastatsis [34, 44]; increasing patient age, tumor size, and extra parenchymal extension are less significant [45]. Lymph node metastasis has been reported more commonly in males than females, is more common in submandibular gland primaries, and develops more frequently in high-grade tumors than low and intermediate grades [45, 46]. Metastases are predictive of poor prognosis and commonly occur in the lung, bone, and brain. Some sites show aggressive behavior regardless of tumor gradings, such as the submandibular gland, which has a prognosis similar to high-grade tumors, tongue, and mouth floor.

## Conclusions

Metastatic MEC to the pleura is exceedingly rare and, to our knowledge, this is the second case reported on this occurrence. Differentiating between a lung primary versus a metastatic disease has treatment implications and prognostic significance for the patient. When morphologic and immunophenotype overlap exists, the molecular diagnosis should be included in the diagnostic arsenal.

## Data Availability

Not applicable.
